# Memorabilia circa Aerem, vitæ genus, sanitatem et Morbos Clausthaliensium

**Published:** 1781-11

**Authors:** 


### LONDON MEDICAL JOURNAL,

For NOVEMBER 1781.

### SECTION I.

### BOOKS.

I. *Memorabilia circa Aerem, vitæ genus, ſanitatem et Morbos Clauſthalienſium. Auctore* L. Fr. Benj. Lentin, M. D. *et Med. Reg. Metall.* 4to. Gottingæ, 1779. p. 144.

THIS work, which ſeems to be the production of a judicious and attentive obſerver, is divided into three ſections. In the firſt ſection, the author treats of the non-naturals. We are told that Clauſthal is ſituated on the Hartz, in a mountainous country, where the weather is extremely variable. The winter ſets in in November with conſiderable ſeverity. The ſummers are cool, but attended with frequent thunder ſtorms, and great falls of rain. The inhabitants, the greateſt part of whom are employed

 either either in the mines or in the founderies, are crouded together in ſmall, heated apartments. Their diet conſiſts of ſalted pork, herrings, puddings, milk, cheeſe, and pulſe. They are ſupplied with pure, wholeſome water, but they drink freely of brandy, and of very indifferent coffee. The miners are expoſed to frequent tranſitions from heat to cold, &c. which ſubject them to a variety of complaints. Thoſe which prevail among the founders are the colic of lead and palſy. The children, who are made to work very early in life and are badly cloathed, are ſubject to orthopnœa, tinea capitis, ſcrophula, bronchocele, epilepſy, and worms, particularly the tænia. The women menſtruate irregularly, and are often troubled with fluor albus.

In the ſecond ſection Dr. Lentin treats of the epidemic diſeaſes of 1774. The ſummer, he obſerves, was cool, ſo that the mercury in Fahrenheit's thermometer ſeldom roſe above 66°. The winter was unuſually cold. The prevailing diſeaſes were putrid and bilious fevers, combined more or leſs with rheumatic affection. Of 779 patients who were intruſted to his care, 22 died. In a table annexed to this part of his work, he gives a ſummary view of the diſeaſes that prevailed amongſt adults and children from 1774 to 1777.

In

In the third and laſt ſection our author ſpeaks of the *ſporadic* diſeaſes that occurred during the ſame period. He informs us that in ſeveral caſes of phthiſis, when the lungs were evidently ulcerated, he experienced good effects from the *oleum petræ*. He confirms the truth of an obſervation made long ago by Hollerius, that violent pains in the calves of the legs, particularly of the right leg, may be reckoned among the ſigns of a diſeaſed liver.

Dr. Lentin is of opinion, that lepra and elephantiaſis always take their riſe from a ſlight cutaneous diſorder. He relates the caſe of a miner, who, after ſitting on the ground in a field during the hemorrhoidal flux, was ſeized with a violent itching of the ſcrotum, which was ſoon ſucceeded by an eruption of puſtules on different parts of the body, but chiefly on the head and genitals. Theſe puſtules were filled with a corroding ichorous fluid, and when the ſcabs were ſcratched off or died away, they left behind them a painful, ſpreading ulcer. Tar water and warm bathing afforded the patient ſome little relief, but at length the diſeaſe brought on hectic fever, which proved fatal.

In the treatment of the colic of lead, Dr. Lentin generally found that emetics, antiſpaſmodics, alkaline ſalts, and purgatives, proved efficacious.

 Warm

Warm bathing and fomentations to the belly were of ufe in mitigating the pain, but he feldom experienced any good effects from opiates.

He obferves that bronchocele is a frequent difeafe in thofe mountainous countries, efpecially in women. When the patient has been under thirty years of age, he has generally found a certain cure in Arnold's *pulvis ad ftrumam*, given in the dofe of a tea-fpoonful three times a day. This powder, if we miftake not, is an empirical remedy, the compofition of which has not yet been made public.

In fpeaking of the hemorrhoids, the author obferves that the *exfudatio hæmorrhoidalis*, as he terms it, produces many difagreeable effects in female patients, by occafioning a morbid irritability, and exciting them to manuftrupation. The Wifbad waters, neutral falts, bleeding, and a milk diet, are recommended as the beft remedies in hæmorrhoidal affection of the bladder.

He fpeaks of a cafe of mania, in which the extract of henbane proved of ufe; and towards the clofe of the work we meet with an account of an emphyfema, produced by a fracture of the cartilages of the third and fifth true ribs, from which the patient recovered.

